# Chinese Clinical Trial Registry 13-year data collection and analysis: geographic distribution, financial support, research phase, duration, and disease categories

**DOI:** 10.3389/fmed.2023.1203346

**Published:** 2023-10-12

**Authors:** Ruitai Fan, Yufei Zheng, Runze Zhou, Narasimha M. Beeraka, Olga A. Sukocheva, Ruiwen Zhao, Shijie Li, Xiang Zhao, Chunying Liu, Song He, P. A. Mahesh, B. M. Gurupadayya, Vladimir N. Nikolenko, Di Zhao, Junqi Liu

**Affiliations:** ^1^Cancer Center, The First Affiliated Hospital of Zhengzhou University, Zhengzhou, China; ^2^Department of Radiation Oncology, The First Affiliated Hospital of Zhengzhou University, Zhengzhou, China; ^3^Raghavendra Institute of Pharmaceutical Education and Research (RIPER), Anantapuramu, Andhra Pradesh, India; ^4^Department of Human Anatomy, I.M. Sechenov First Moscow State Medical University (Sechenov University), Moscow, Russia; ^5^Department of Pediatrics, Herman B. Wells Center for Pediatric Research, Indiana University School of Medicine, Indianapolis, IN, United States; ^6^College of Nursing and Health Sciences, Flinders University of South Australia, Bedford Park, SA, Australia; ^7^College of Medicine, Zhengzhou University, Zhengzhou, China; ^8^Department of Pulmonary Medicine, JSS Medical College, JSS Academy of Higher Education and Research (JSS AHER), Mysuru, Karnataka, India; ^9^Department of Pharmaceutical Chemistry, JSS College of Pharmacy, JSS Academy of Higher Education and Research (JSS AHER), Mysuru, Karnataka, India; ^10^Department of Endocrinology, The First Affiliated Hospital of Zhengzhou University, Zhengzhou, China

**Keywords:** Chinese Clinical Trial Registry, year-on-year China, health information, management and policy, governmental registry

## Abstract

**Objective:**

To evaluate the current status of trial registration on the Chinese Clinical Trial Registry (ChiCTR).

**Design:**

In this descriptive study, a multi-dimensional grouping analysis was conducted to estimate trends in the annual trial registration, geographical distribution, sources of funding, targeted diseases, and trial subtypes.

**Setting:**

We have analyzed all clinical trial records (over 30,000) registered on the Chinese Clinical Trial Registry (ChiCTR) from 2007 to 2020 executed in China.

**Main outcome(s) and measure(s):**

The main outcome was the baseline characteristics of registered trials. These trials were categorized and analyzed based on geographical distribution, year of implementation, disease type, resource and funding type, trial duration, trial phase, and the type of experimental approach.

**Results:**

From 2008 to 2017, a consistent upward trend in clinical trial registrations was observed, showing an average annual growth rate of 29.2%. The most significant year-on-year (yoy%) growth in registrations occurred in 2014 (62%) and 2018 (68.5%). Public funding represented the predominant source of funding in the Chinese healthcare system. The top five ChiCTR registration sites for all disease types were highly populated urban regions of China, including Shanghai (5,658 trials, 18%), Beijing (5,127 trials, 16%), Guangdong (3,612 trials, 11%), Sichuan (2,448 trials, 8%), and Jiangsu (2,196 trials, 7%). Trials targeting neoplastic diseases accounted for the largest portion of registrations, followed by cardio/cerebrovascular disease (CCVD) and orthopedic diseases-related trials. The largest proportions of registration trial duration were 1–2 years, less than 1 year, and 2–3 years (at 27.36, 26.71, and 22.46%). In the case of the research phase, the top three types of all the registered trials are exploratory research, post-marketing drugs, and clinical trials of new therapeutic technology.

**Conclusion and relevance:**

Oncological and cardiovascular diseases receive the highest share of national public funding for medical clinical trial-based research in China. Publicly funded trials represent a major segment of the ChiCTR registry, indicating the dominating role of public governance in this health research sector. Furthermore, the growing number of analyzed records reflect the escalation of clinical research activities in China. The tendency to distribute funding resources toward exceedingly populated areas with the highest incidence of oncological and cardiovascular diseases reveals an aim to reduce the dominating disease burden in the urban conglomerates in China.

## Highlights

-Question: What are the current characteristics of clinical trials registered on the Chinese Clinical Trial Registry (ChiCTR) platform in the last 13 years?

-Findings: In this descriptive research, clinical trials pertinent to neoplastic diseases occupied the largest portion of registrations. Publicly funded trials accounted for a comparatively higher number than the trials funded by other sources. Research on oncological and cardiovascular diseases receives the highest share of national public funding for medical research when compared to other types of disease research.

-Meaning: This study provides significant insights and background for the development of regulations that promote greater transparency and accuracy in clinical trial registrations and research distribution.

## Background

### Database description of data acquisition from ChiCTR.gov

Clinical trials are registered and analyzed to provide public health researchers, medical staff, and trial sponsors with reliable and authentic information regarding the trial implementation, as well as the ability for oversight and transparency (1, 2). The Chinese Clinical Trial Registry (ChiCTR) was launched in 2007, as per the guidelines described by the International Clinical Trials Register (CTR) Platform Standards and the Ottawa Group Standards. Currently, ChiCTR is the third largest CTR in the world preceded only by ClinicalTrials.gov and the EU Clinical Trials Register (3–5). Clinical research reports in this registry have been updated when required. Accordingly, ChiCTR re-structuring was facilitated by several sponsors and researchers who are involved in determining subject matter and trial planning (6). International cooperation and multi-center research applications are supported by the current CTR protocols.

In this study, the records of all the registered trials from 2007 to 2020 were retrieved from ChiCTR. Using the retrieved data, we aimed to identify and analyze key trial characteristics, including targeted diseases and pathologies. Our data analysis provides insights into the state of clinical research in China, the geographical distribution of trial registration, the registered duration of trials, the research phase, the type of experimental approach, and the funding types reported in a 13-year duration (2007–2020).

## Methods

### Data collection

In China, all medical investigations and clinical trials must be registered on the ChiCTR platform in accordance with the Declaration of Helsinki (2008) and the Regulations of China Food and Drug Administration (CFDA). The records were extracted from the ChiCTR platform in batches from September 5 to October 21, 2020. Finally, the screened clinical trials were included in a research database using Microsoft Excel for further analysis.

We manually retrieved baseline characteristics from each trial record including trial title, geographic location, funding and material sources, disease type, methodological design, implementation period, duration, and research phase. The methodological design of a trial was assessed considering its dominant contribution toward the effectiveness of investigations. Registered trial types were as follows: interventional, observational, etiological, diagnostic, basic, and epidemiological studies, assessing new drugs and non-drug treatments, preventive, prognostic, screening, and health service-related interventions. The funding type was also assessed as a contributing factor that can influence data reporting and conclusion bias (7–9).

### Categorization by geographic location, disease, funding, research phase, and duration of trials

The registered clinical trials were categorized according to the geographic location of the head units, implementation time (year), resources and funding type (public, industry, self-sponsored, or funding with no specified sponsors), experimental study design (interventional, observational, epidemiological, case–control, and other types), and disease type. The top five disease-related categories included oncological (1), cardiovascular and/or cerebrovascular (2), orthopedic (3), gastroenterological (4), and respiratory (5) diseases. Information related to the duration of trials (less than 1 year, 1–2 years, 2–3 years, 3–4 years, 4–5 years, and more than 5 years), 1,719 trials were missed and were defined as NA (Not available). Information pertinent to the research phase of each trial was obtained depending on phase I, phase II, phase III, phase IV, retrospective trial, and other phases.

Trials, initiated before the year 2007, were merged into the “2007 or earlier” group. Small-scale trials that targeted <1% of the total sample size (≤320 cases) were merged into “others” (112 screening trials, 149 prevention trials, 222 prognostic trials, and 153 medical service trials) for the convenience of data analysis. Trials that involved the analysis of two or more diseases were classified as “multi-disciplinary trials.” Altogether, 472 trials were not accompanied by the relevant disease or pathology-based description and were classified as the “NA” group.

“Public funding” was defined as the acquisition of financial sources from government sources, colleges, universities, non-profit medical organizations, and other scientific institutions worldwide. “Industry funding” indicates the acquisition of resources from corporations, business associations, and any other for-profit organizations. The “self-sponsored” category indicates that the funding resources were from the experimenter himself or belonging to a group. The “No funding” category includes trials that were executed without any identified form of funding.

### Data analysis

We calculated the year-on-year growth rate (yoy%) of trial numbers registered on the ChiCTR platform to describe the annual variation trend of ChiCTR trials. The percentage composition of different types of registered trials was calculated. The disease types were ranked and presented using a horizontal bar chart. A gradual color-coded map was used to present the CTR geographic distribution in China. To visualize the status of funding type for different trial types from multiple prospects, we calculated and described the distribution of funding sources using the annual variation trends and the trends in the percentage of trials funded per year. Furthermore, we analyzed these variables for the following top five disease types: oncological, cardio- and cerebrovascular, orthopedic, gastroenterological, and respiratory diseases.

## Results

Following the data extraction procedures, a total of 32,551 clinical trial records were found to be registered before October 17, 2020 on the ChiCTR platform. We performed data processing and examination procedures. We excluded trial records associated with the missing administration spot information (359 records), trial records registered as “the chief unit is located outside of China” (148 records), and duplicated records as per the trial registration number (27 records). Finally, we included 32,017 clinical trials registered on ChiCTR in the research database. Detailed information about these trials and their baseline characteristics was presented in Table 1 and summarized the clinical trial disease distribution by dividing the trial records into 22 groups. Neoplastic disease trials were separated into distinct categories due to their significant impact on public health services. Addressing the outbreak of SARS-CoV-2 (severe acute respiratory syndrome coronavirus 2), trials concerning COVID-19 were also merged into a separate group. Later, we executed the data screening and deduplication processes; and we included 32,017 trials in our final analysis. The interventional trials accounted for more than half of the total trials (17,771 trials, 56%), followed by observational (7,701 trials, 24%), etiology-addressing (2,195 trials, 7%), and diagnostic trials (1,783 trials, 6%).

**Table 1:** The characteristics and summary of all the trials registered on ChiCTR platform.

Item	Characteristics	Number (%) of trials with characteristics
Year		
	2007 or earlier	481 (1.5)
	2008	332 (1)
	2009	416 (1.3)
	2010	530 (1.7)
	2011	614 (1.9)
	2012	781 (2.4)
	2013	976 (3)
	2014	1,581 (4.9)
	2015	1822 (5.7)
	2016	2,360 (7.4)
	2017	3,288 (10.3)
	2018	5,541 (17.3)
	2019	6,073 (19)
	2020	6,374 (19.9)
	NA	848 (2.6)
Category		
	Basic	1,026 (3.2)
	Diagnostic	1783 (5.6)
	Epidemiological	362 (1.1)
	Etiology	2,195 (6.9)
	Interventional	17,771 (55.5)
	Observational	7,701 (24.1)
	Others	636 (2)
	Treatment	353 (1.1)
	NA	190 (0.6)
Funding type		
	Industry	2,691 (8.4)
	No_sponsor	2091 (6.5)
	Public	19,828 (61.9)
	Self_funding	6,935 (21.7)
	NA	472 (1.5)
Disease		
	Anesthesiology	882 (2.8)
	Cardio-cerebral vascular diseases	4,468 (14)
	COVID-19	676 (2.1)
	Dermatology	363 (1.1)
	Endocrinology	1,612 (5)
	Epidemiology	1,243 (3.9)
	Gastroenterology	2,152 (6.7)
	Hematology	626 (2)
	Imageology	217 (0.7)
	Multi-disciplinary	928 (2.9)
	Neurology	1,579 (4.9)
	Nursing	116 (0.4)
	Oncology	5,637 (17.6)
	Ophthalmology	1,031 (3.2)
	Orthopedics	2,535 (7.9)
	Otolaryngology	330 (1)
	Procreation	1,633 (5.1)
	Psychology	773 (2.4)
	Rehabilitation	288 (0.9)
	Respiratory	1780 (5.6)
	Rheumatic immunology	676 (2.1)
	Stomatology	428 (1.3)
	Urology	956 (3.0)
	Others	1,088 (3.4)
Geographical distribution*		
	North China	6,504 (20.3)
	Northeast China	1,213 (3.8)
	East China	12,245 (38.2)
	South Central China	6,309 (19.7)
	Southwest region	4,194 (13.1)
	Northwest territories	1,078 (3.4)
	Hong Kong and Marco	474 (1.5)

*The definition of geographical division is in accordance with the six administrative divisions provided by the Central People’s government of the PRC website.

### Analysis of annual trend and geographic distribution

**Figure 1:**
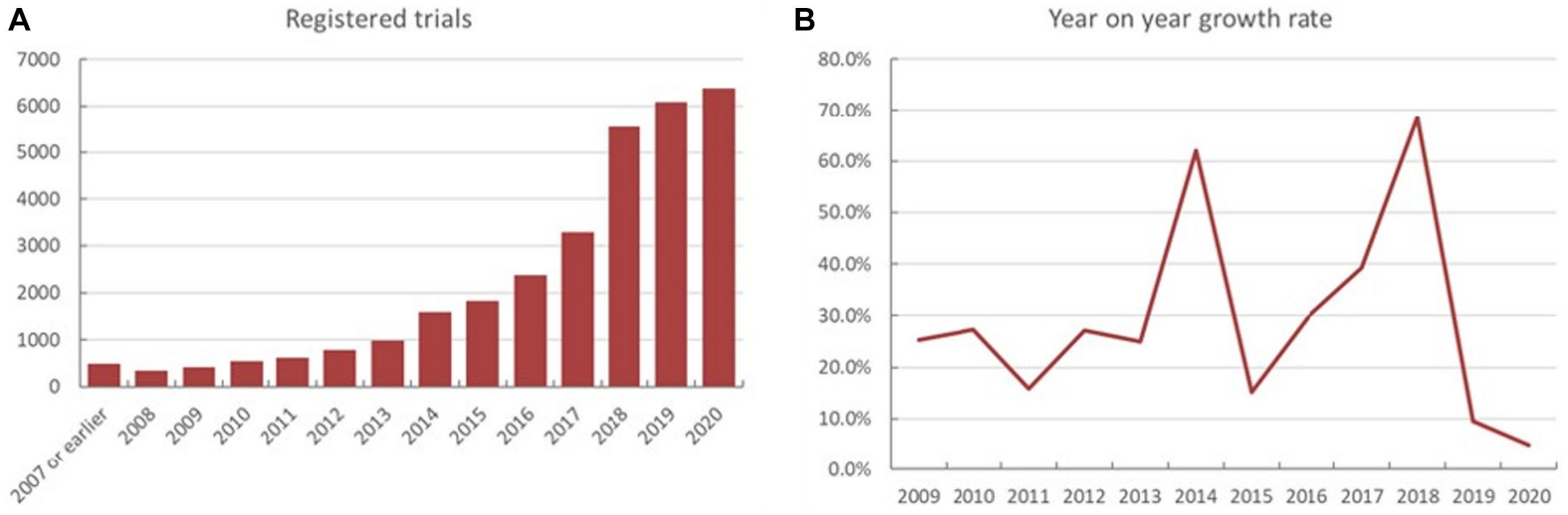
The yearly fluctuations in both the exact number **(A)** and year-on-year percentage (yoy%) on the ChiCTR platform **(B)**.

Analysis of the registered trials indicated that 481 trials were registered during 2007 or earlier. The numerical trend of increase in the registered trial number varied from year to year (Figures 1A,B). Total number of ChiCTR trials was steadily growing upwards during the period of 2008 to 2017.Total number of registrations during the period of 2017 to 2018 substantially increased from 3,288 to 5,541 (yoy% growth at 68.5%), which later returned to a relatively steady upward trend.

**Figure 2:**
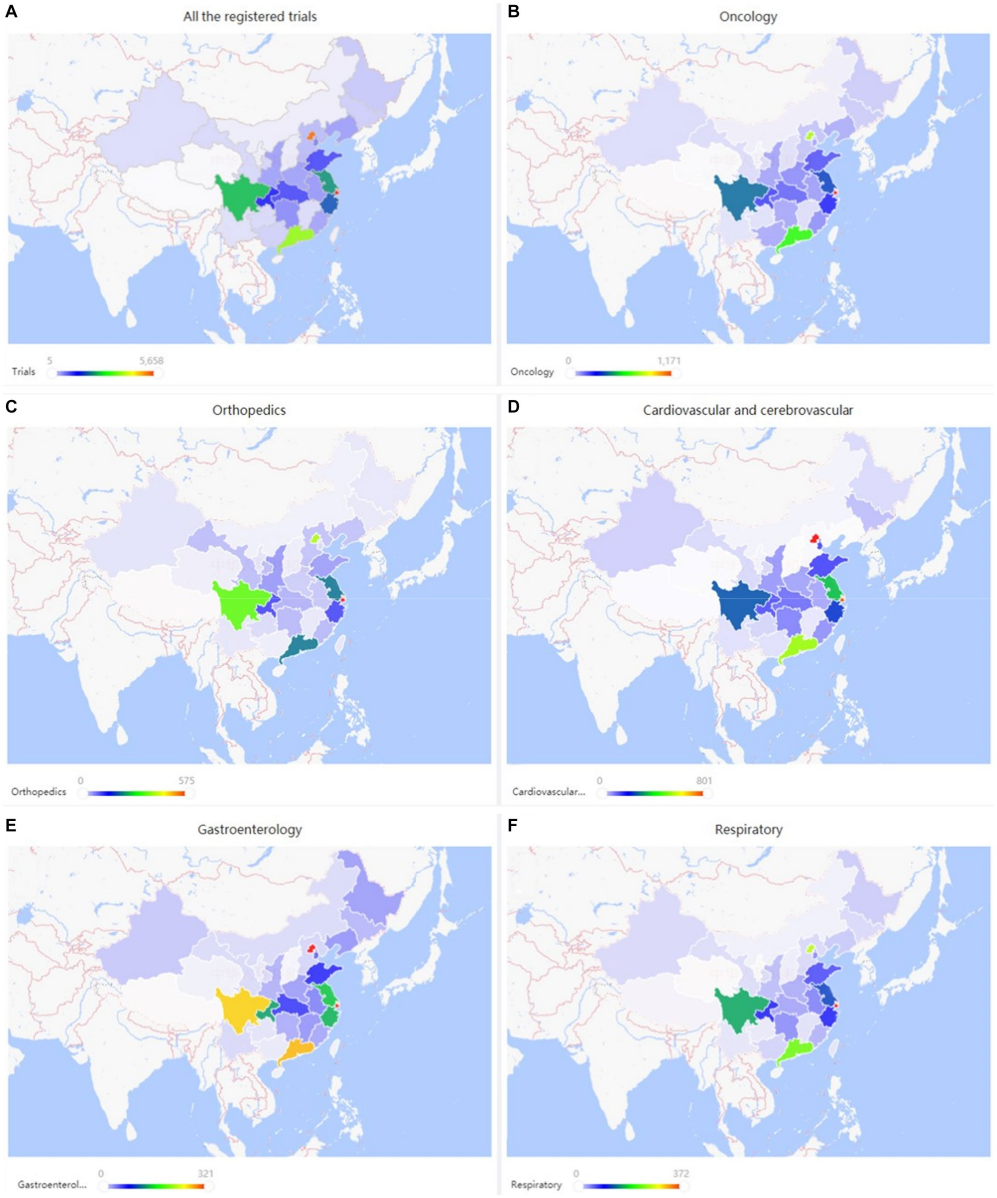
**(A–F)** Geographical distribution of registered trials for overall and top three disease/pathology types.

The geographic *distribution of registered trials* is shown in Figures 2A–F. A higher number of trial registrations was observed in Southeast China. The number of trial registrations decreased gradually westward. Shanghai (5,658 trials, 18%) (Table 2), Beijing (5,127 trials, 16%), Guangdong (3,612 trials, 11%), Sichuan (2,448 trials, 8%), and Jiangsu (2,196 trials, 7%) were ranked among the top five provinces with the highest number of trial registration for all disease types in China.

**Table 2:** Geographical distribution of registered trials for overall and top five disease/pathology types.

Region	Trials (%)	Oncology (%)	Cardiovascular and cerebrovascular (%)	Orthopedics (%)	Gastroenterology (%)	Respiratory (%)
Shanghai	5,658 (17.7%)	1,171 (20.8%)	740 (16.6%)	385 (15.2%)	321 (14.9%)	254 (14.9%)
Beijing	5,127 (16.0%)	784 (13.9%)	801 (17.9%)	575 (22.7%)	317 (14.7%)	372 (14.7%)
Guangdong	3,612 (11.3%)	621 (11.0%)	521 (11.7%)	207 (8.2%)	255 (11.8%)	153 (11.8%)
Sichuan	2,448 (7.6%)	407 (7.2%)	263 (5.9%)	206 (8.1%)	143 (6.6%)	114 (6.6%)
Jiangsu	2,196 (6.9%)	358 (6.4%)	351 (7.9%)	336 (13.3%)	263 (12.2%)	224 (12.2%)
Zhejiang	1809 (5.7%)	269 (4.8%)	236 (5.3%)	112 (4.4%)	136 (6.3%)	86 (6.3%)
Chongqing	1,360 (4.2%)	230 (4.1%)	142 (3.2%)	46 (1.8%)	53 (2.5%)	45 (2.5%)
Shandong	1,109 (3.5%)	208 (3.7%)	153 (3.4%)	59 (2.3%)	66 (3.1%)	35 (3.1%)
Hubei	1,100 (3.4%)	187 (3.3%)	118 (2.6%)	21 (0.8%)	33 (1.5%)	19 (1.5%)
Tianjin	780 (2.4%)	111 (2.0%)	154 (3.4%)	112 (4.4%)	130 (6.0%)	86 (6.0%)
Hunan	714 (2.2%)	109 (1.9%)	102 (2.3%)	42 (1.7%)	28 (1.3%)	45 (1.3%)
Anhui	634 (2.0%)	119 (2.1%)	73 (1.6%)	29 (1.1%)	12 (0.6%)	7 (0.6%)
Liaoning	595 (1.9%)	101 (1.8%)	0 (0.0%)	0 (0.0%)	0 (0.0%)	3 (0.0%)
Shanxi	583 (1.8%)	113 (2.0%)	84 (1.9%)	36 (1.4%)	26 (1.2%)	25 (1.2%)
Henan	578 (1.8%)	139 (2.5%)	75 (1.7%)	62 (2.4%)	72 (3.3%)	69 (3.3%)
Fujian	558 (1.7%)	137 (2.4%)	90 (2.0%)	33 (1.3%)	30 (1.4%)	29 (1.4%)
Xianggang	469 (1.5%)	36 (0.6%)	63 (1.4%)	42 (1.7%)	42 (2.0%)	48 (2.0%)
Hebei	405 (1.3%)	70 (1.2%)	2 (0.0%)	12 (0.5%)	18 (0.8%)	14 (0.8%)
Heilongjiang	329 (1.0%)	60 (1.1%)	29 (0.6%)	8 (0.3%)	11 (0.5%)	4 (0.5%)
Jilin	289 (0.9%)	61 (1.1%)	52 (1.2%)	22 (0.9%)	13 (0.6%)	14 (0.6%)
Guangxi	264 (0.8%)	104 (1.8%)	23 (0.5%)	39 (1.5%)	36 (1.7%)	20 (1.7%)
Gansu	219 (0.7%)	39 (0.7%)	30 (0.7%)	11 (0.4%)	12 (0.6%)	8 (0.6%)
Guizhou	210 (0.7%)	33 (0.6%)	33 (0.7%)	12 (0.5%)	8 (0.4%)	7 (0.4%)
Jiangxi	197 (0.6%)	37 (0.7%)	17 (0.4%)	11 (0.4%)	2 (0.1%)	5 (0.1%)
Xinjiang	196 (0.6%)	39 (0.7%)	38 (0.9%)	35 (1.4%)	33 (1.5%)	32 (1.5%)
Yunnan	164 (0.5%)	29 (0.5%)	23 (0.5%)	20 (0.8%)	24 (1.1%)	13 (1.1%)
Shanxi	105 (0.3%)	33 (0.6%)	0 (0.0%)	0 (0.0%)	0 (0.0%)	0 (0.0%)
Neimenggu	87 (0.3%)	15 (0.3%)	7 (0.2%)	12 (0.5%)	36 (1.7%)	10 (1.7%)
Taiwan	84 (0.3%)	3 (0.1%)	9 (0.2%)	14 (0.6%)	6 (0.3%)	16 (0.3%)
Ningxia	65 (0.2%)	9 (0.2%)	7 (0.2%)	8 (0.3%)	3 (0.1%)	9 (0.1%)
Hainan	41 (0.1%)	4 (0.1%)	5 (0.1%)	6 (0.2%)	3 (0.1%)	2 (0.1%)
Qinghai	15 (0.0%)	1 (0.0%)	1 (0.0%)	13 (0.5%)	11 (0.5%)	12 (0.5%)
Xizang	12 (0.0%)	0 (0.0%)	1 (0.0%)	2 (0.1%)	3 (0.1%)	0 (0.1%)
Aomen	5 (0.0%)	0 (0.0%)	1 (0.0%)	7 (0.3%)	6 (0.3%)	0 (0.3%)

**Figure 3:**
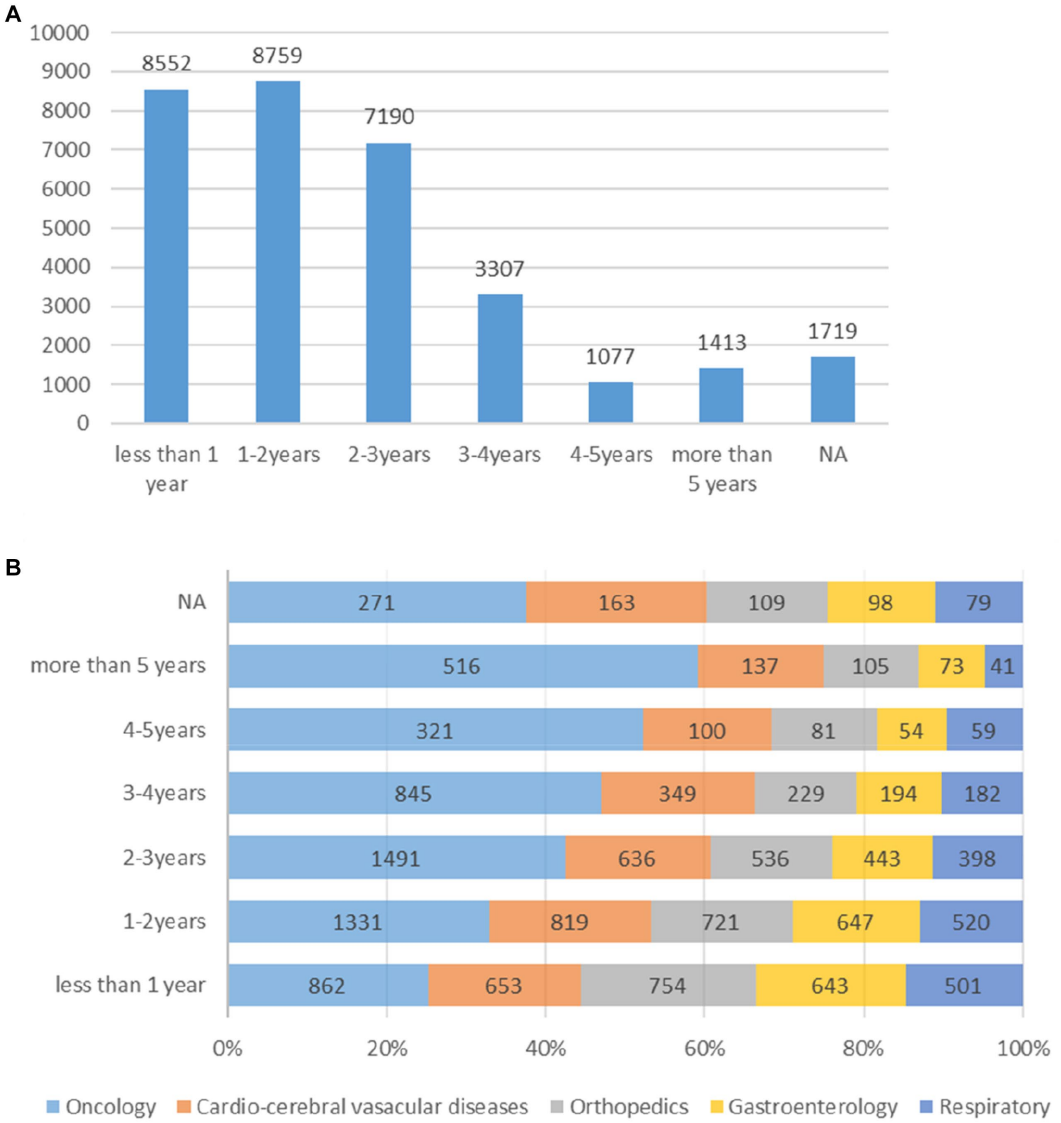
**(A,B)** Analysis of information related to the duration of registered trials overall and the top five diseases/pathology types.

Duration of each registered trial based was examined on the starting and ending time spots. Overall, trials for 1–2 years accounted for the largest part (8,759 trials, 21.36%) (Figure 3A), followed by trials for less than 1 year (8,552 trials, 26.71%), trials for 2–3 years (7,190 trials), trials for 3–4 years (3,307 trials, 10.33%) and trials for more than 5 years (1,413 trials, 4.41%). The 4–5 years trials composed the lowest part (1,077 trials, 3.36%, Table 1).

**Figure 4:**
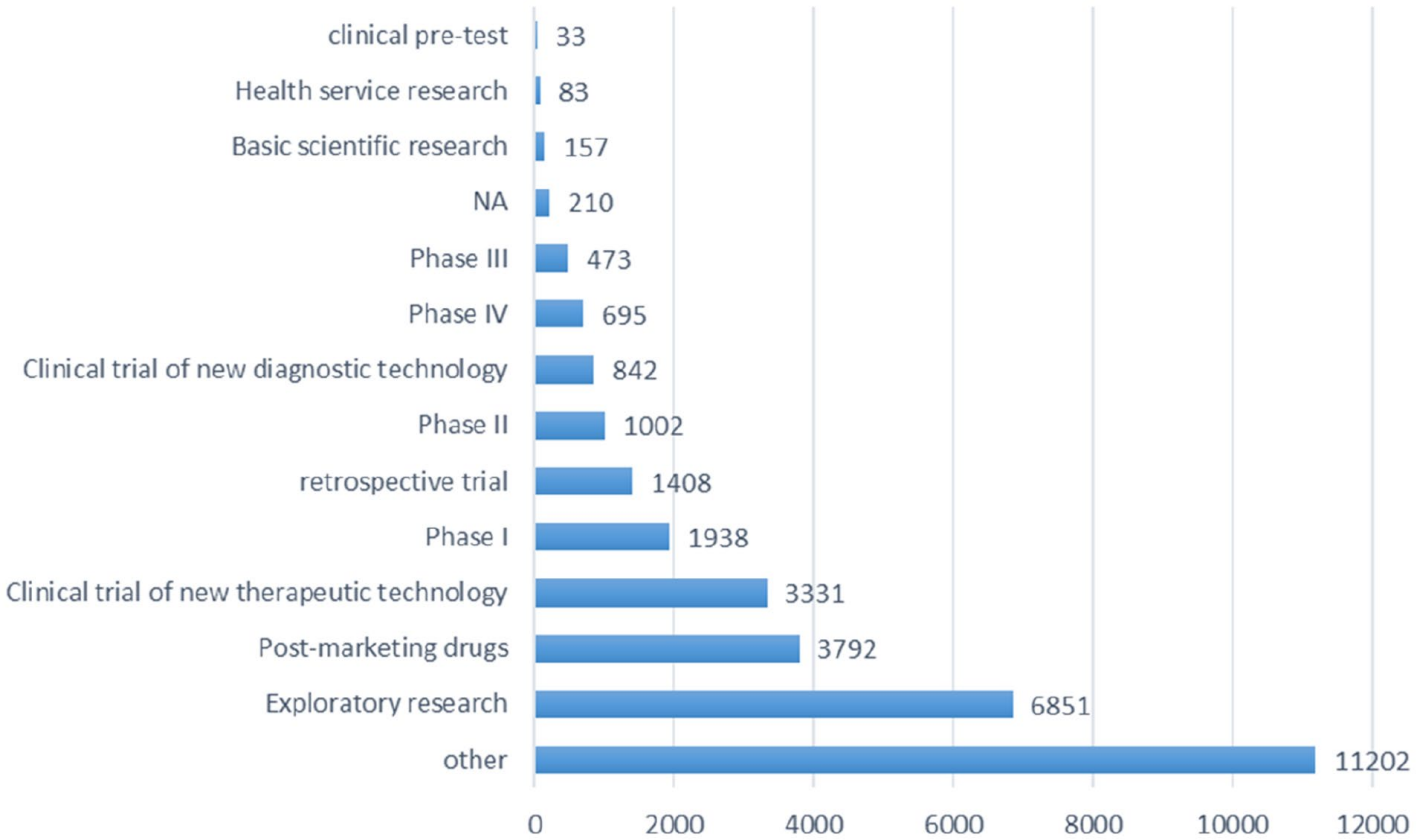
Schematic depiction of the information analysis pertinent to the research phase of all registered trials.

The *research phase* records of registered trials were divided into 14 groups (Figure 4), including exploratory research (6,851 trials, 21.40%), post-marketing drugs (3,792 trials, 11.84%), clinical trial of new therapeutic technology (3,331 trials, 10.40%), phase I (1938 trials, 6.05%), retrospective trial (1,408 trials, 4.40%) and other types.

### Analysis of disease type

**Figure 5:**
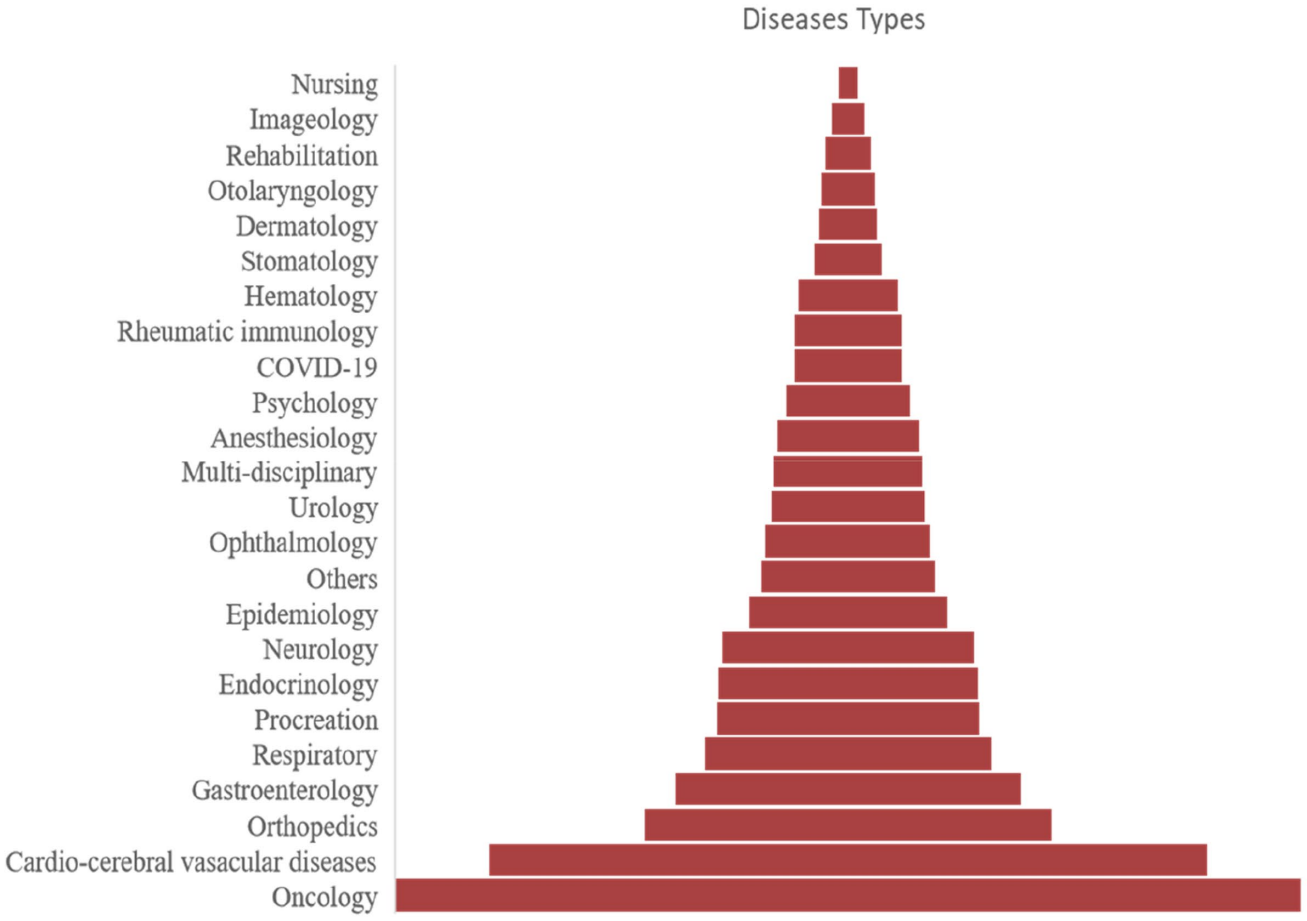
Analysis of disease/pathology distribution in ChiCTR Database.

Oncology-related trials accounted for the largest portion of total trials (5,637 trials, 17.6%) (Table 1), followed by trials targeting CCVD (4,468 trials, 14.0%), orthopedic diseases (2,535 trials, 7.9%), gastroenterology diseases (2,152 trials, 6.7%), and respiratory diseases (1,780 trials, 5.6%). The distribution of all the registered trials across *different disease types* was shown in Figure 5. Other than the top 5 disease-targeting trials, a total of 676 trials (2.1%) SARS-CoV-2-targeting trials represented a significant proportion of studies organized for the last 2 years.

The trials that involved the analysis of two or more types of diseases were classified as “multi-disciplinary” (928 trials, 2.9%). The top three disease types were presented with the aspect of the annual trend, geographic distribution, proportion, and registered duration in the following part.

### Oncological diseases trials

**Figure 6:**
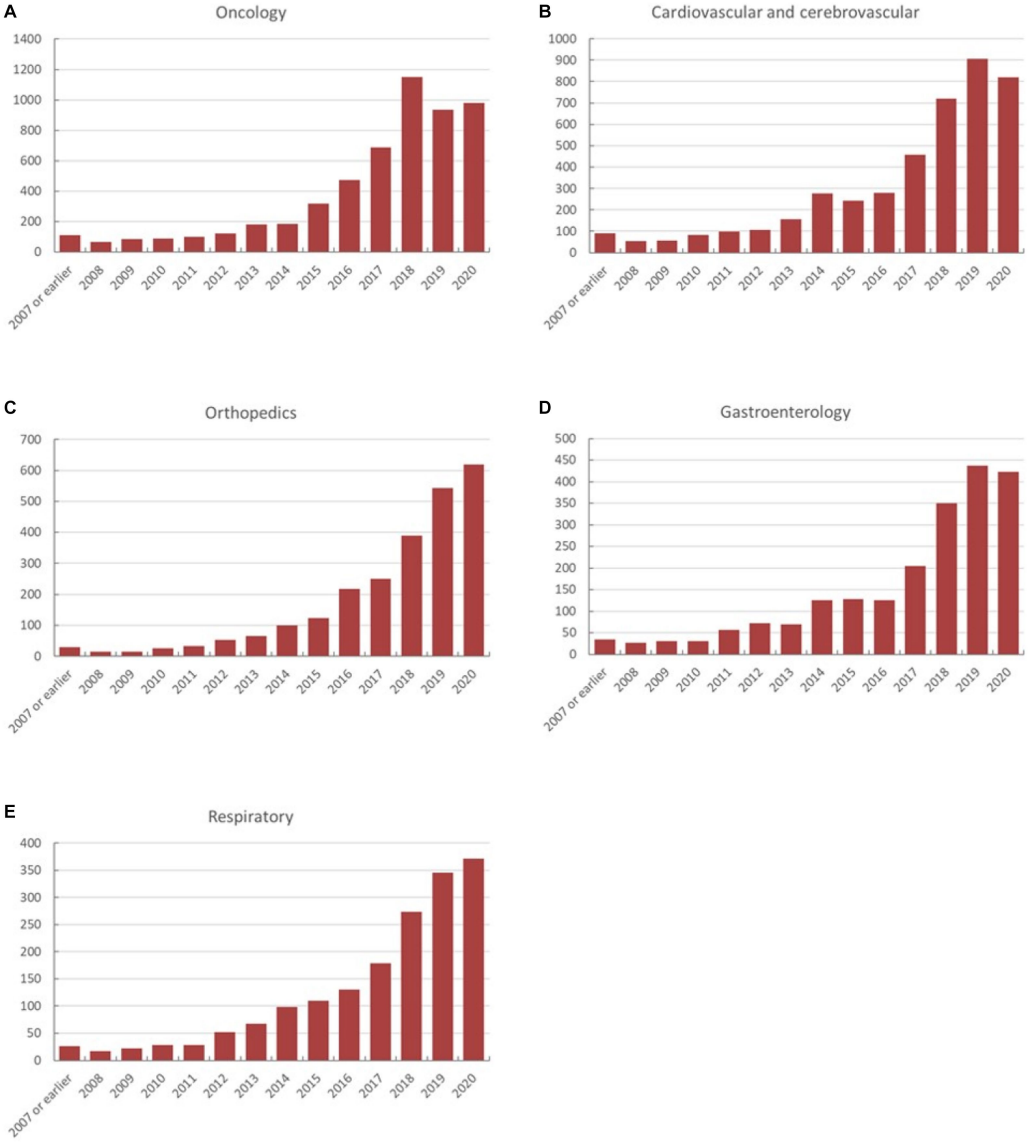
**(A–E)** The annual variation of registered trials on ChiCTR is categorized according to the disease/pathology type.

Neoplastic diseases account for the largest part (5,637 trials, 17.6%) of all registered studies in ChiCTR. The trend in neoplastic disease-targeting trials was shown in Figure 6. Higher growth in the number of trials was observed during the period 2014 to 2020 with an average 31% increase in yoy. A flat trend was observed before 2014. The geographic distribution of neoplastic disease targeting trials was shown in Figure 2B and Table 2. The number of trials in Shanghai (1,171 trials, 21%), Beijing (784 trials, 14%), Guangdong (621 trials, 11%), Sichuan (407 trials, 7%), and Jiangsu (358 trials, 6%) ranked the top 5 regions for neoplastic diseases trials in ChiCTR.

**Figure 7:**
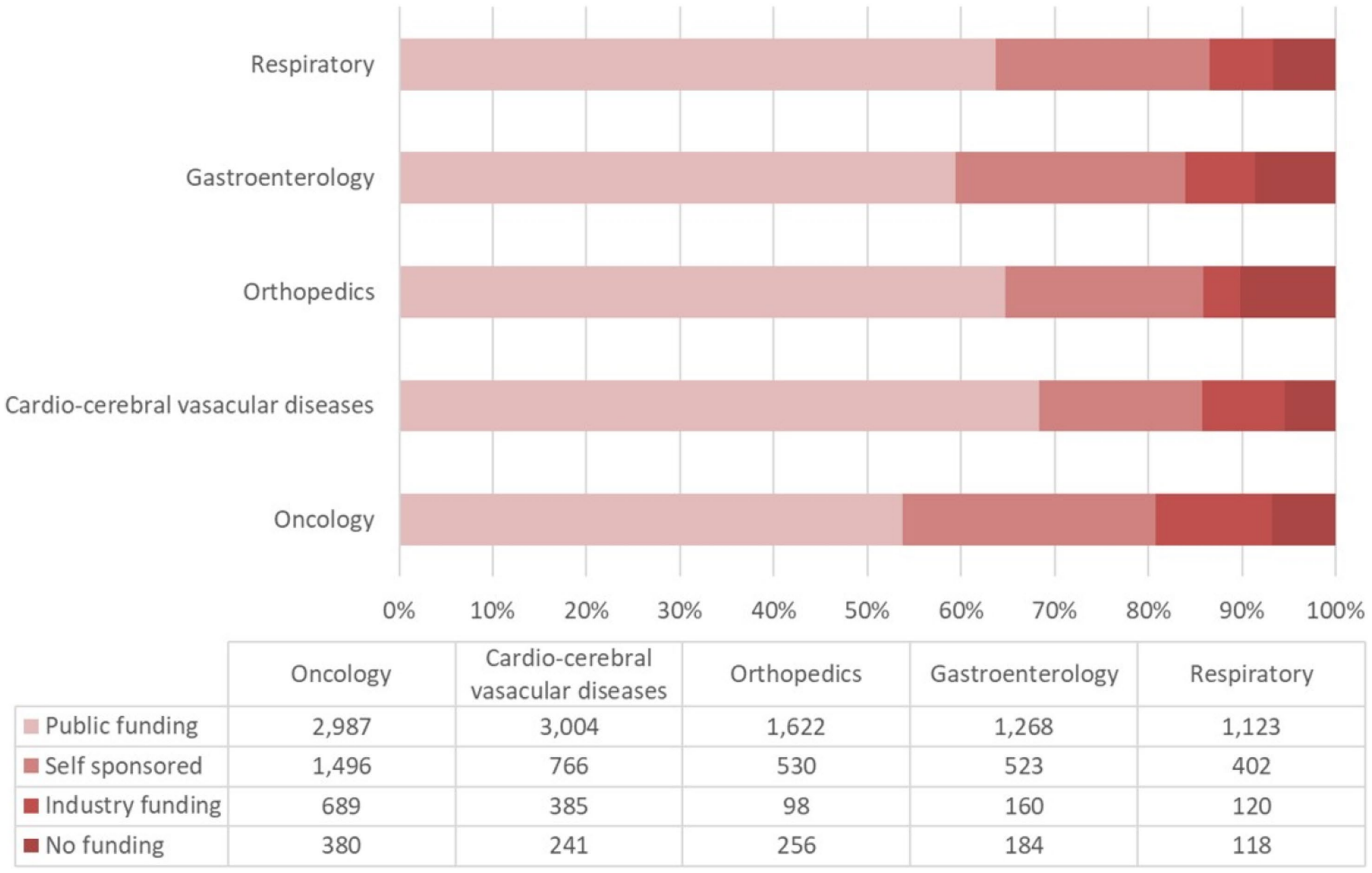
The proportions of financial support types for the top five addressed diseases/pathologies in ChiCTR.

**Figure 8:**
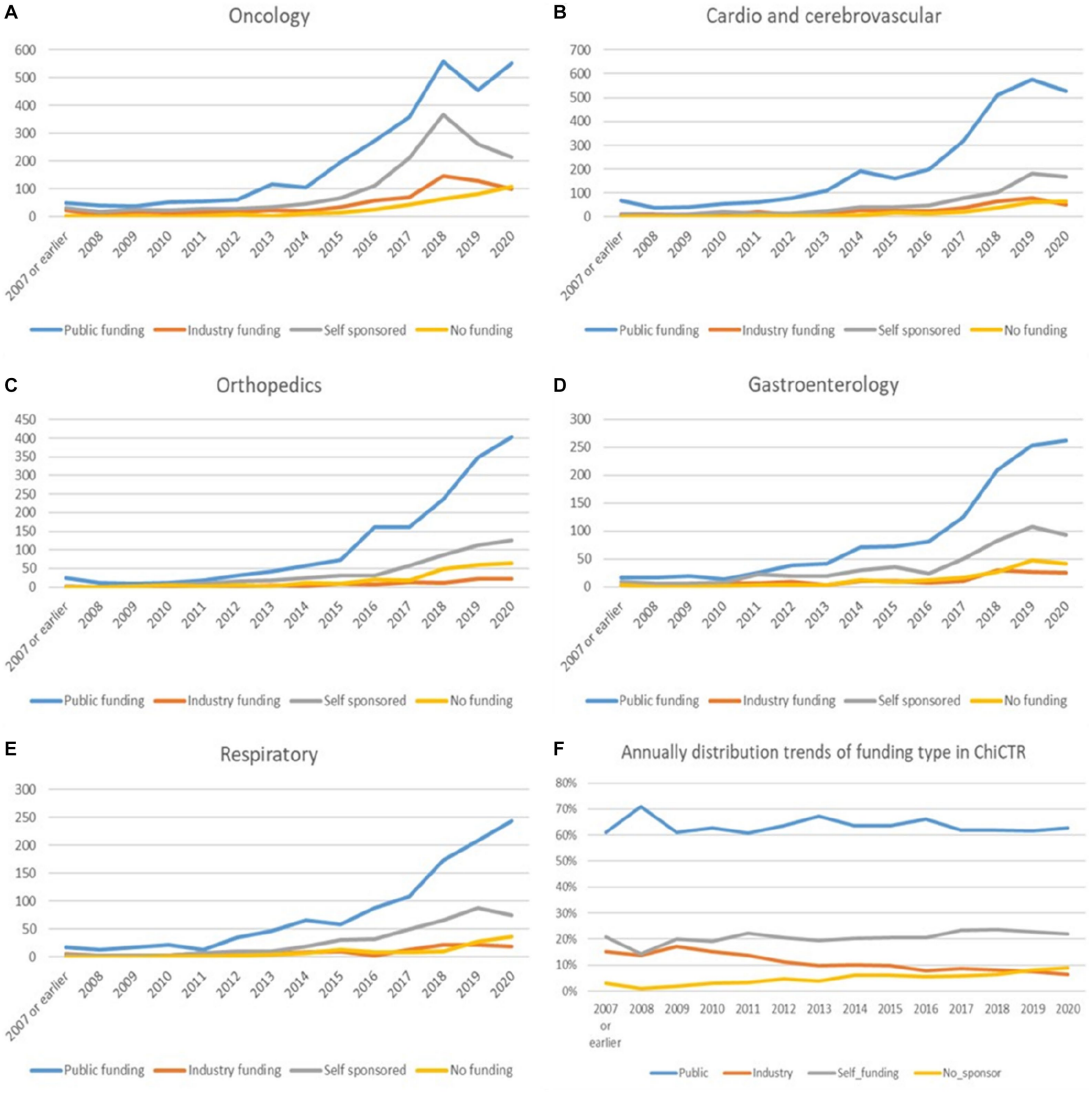
**(A–F)** The annual trends in funding types for the top three diseases/pathologies and yearly variation of the percentage for each funding type in the past 13 years.

The proportion of each funding source for oncological trials is presented in Figure 7. Trials funded by public funding sources composed the largest percentage of oncological disease trials (2,987 trials, 53% of the total trials), followed by self-sponsored support (1,496 trials, 27%), industry funding (689 trials, 12%), and no-funding group (380 trials, 7%). The funding growth was relatively consistent for each funding type with the overall growth trend toward neoplastic disease trials (Figure 8A). The number of registered trials in the no-funding category demonstrated a stable overall increase without variations during 2018–2019. The growth of oncological disease trials showed a relatively consistent and upward trend in each funding type until 2018, then meeting an obvious decrease in 2019.

In case of the duration of trial, 2–3 years occupied the largest percentage of oncological disease trials (1,491 trials, 26.45% of the oncology disease), followed by 1–2 years (1,331 trials, 23.61%), less than 1 year (862 trials,15.29%), 3–4 years (845 trials, 14.99%), more than 5 years (516 trials, 9.15%), 4–5 years (321 trials, 5.69%) respectively (Figure 3B).

### Cardio/−cerebrovascular diseases trials

Trials registered to target cardio/−cerebrovascular diseases (CCVD) accounted for 14% of the total sample (4,468 trials). The CCVD field trend was shown in Figure 6B. The CCVD trial number was on a moderate rise until 2013 with an average yoy% growth of 25%. The highest yoy% growth occurred in 2014 (78%) and 2017 (64%). The geographic distribution of CCVD-registered trials was shown in Figure 2D and Table 2. Beijing (801 trials, 18%), Shanghai (740 trials, 17%), Guangdong (521 trials, 12%), Jiangsu (351 trials, 8%), and Sichuan (263 trials, 6%) ranked in the top 5 regions in CCVD field trial registration.

More than half of all CCVD trials were funded by public sources (3,004 trials, 67%) (Figure 7). The second largest group (17%) of CCVD trials was self-funded (766 trials). A total of 385 trials received industry funding (9%) and 241 trials (5%) received no funding.

The duration of CCVD were as follows: 1–2 years (819 trials, 28.67%) (Figure 3B) composed the largest part of all the CCVD-related trials, followed by less than 1-year trials (653 trials, 22.86%), 2–3 years (636 trials, 22.26%), 3–4 years (349 trials, 12.22%), more than 5 years (137 trials, 4.80%) and 4–5 years (100 trials, 3.50%).

### Orthopedics trials

There were 2,535 trials registered to address orthopedic diseases, accounting for 7.9% of all registered trials, ranking third among ChiCTR clinical trials. The number of trials in orthopedics has been gradually increasing with an average yoy% at 38% within the analyzed period from 2009 to 2020. Two peak yoy% increases were observed in 2011 (73%) and 2016 (75%) (Figure 6C). The geographic distribution of orthopedics-targeting trials was shown in Figure 2C, and summarized in Table 2. Beijing (575 trials, 23%), Shanghai (385 trials, 15%), Jiangsu (336 trials, 13%), Guangdong (207 trials, 8%), and Sichuan (206 trials, 8%) were the top 5 areas with trials in this field. Public funding sources supported 64% of orthopedics-related trials (1,622 trials) (Figure 7). This group was followed by self-sponsored (530 trials, 21%), no-funding (256 trials, 10%), and industry-funded (98 trials, 4%) trials. The variation in funding trend was shown in Figure 8C. We also depicted the distribution of the duration of orthopedic diseases in Figure 3B, including trials: less than 1 year (754, 29.74%), 1–2 years (721, 28.44%), 2–3 years (536, 21.14%), 3–4 years (229, 9.03%), NA (109, 4.30%), more than 5 years (105, 4.14%), 4–5 years (81, 3.20%).

### Analysis of source and funding

**Figure 9:**
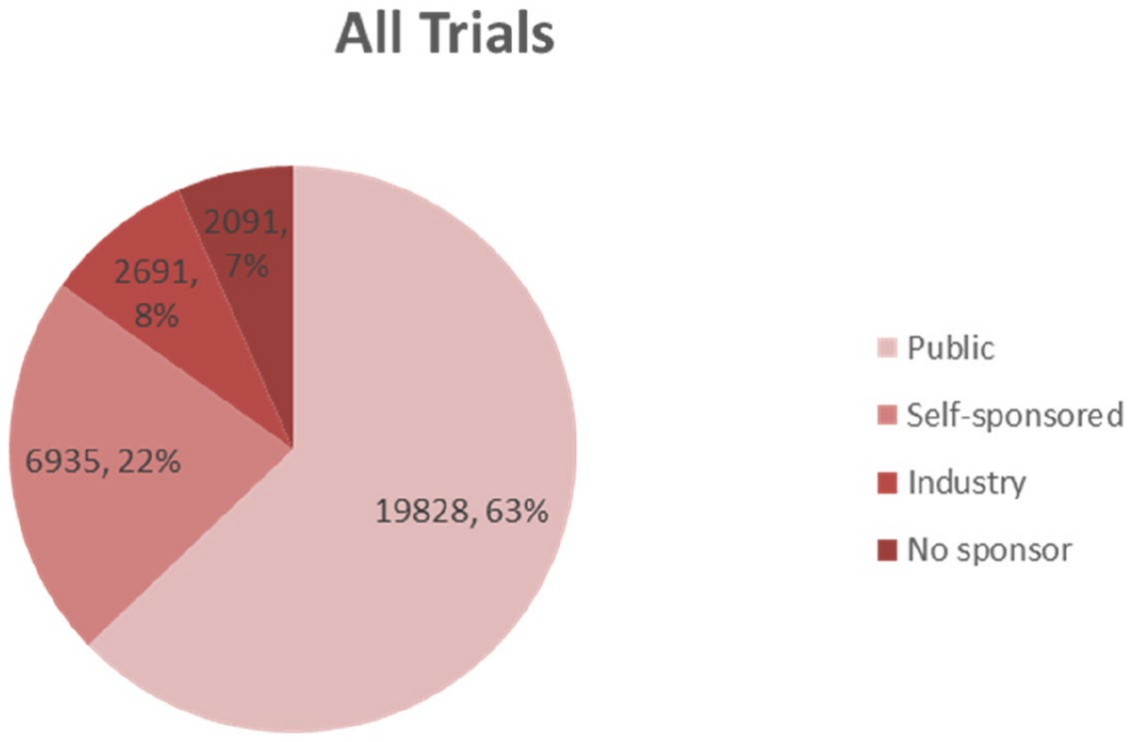
The overall proportion for each type of funding indicates that public funding accounts for the highest part.

**Figure 10:**
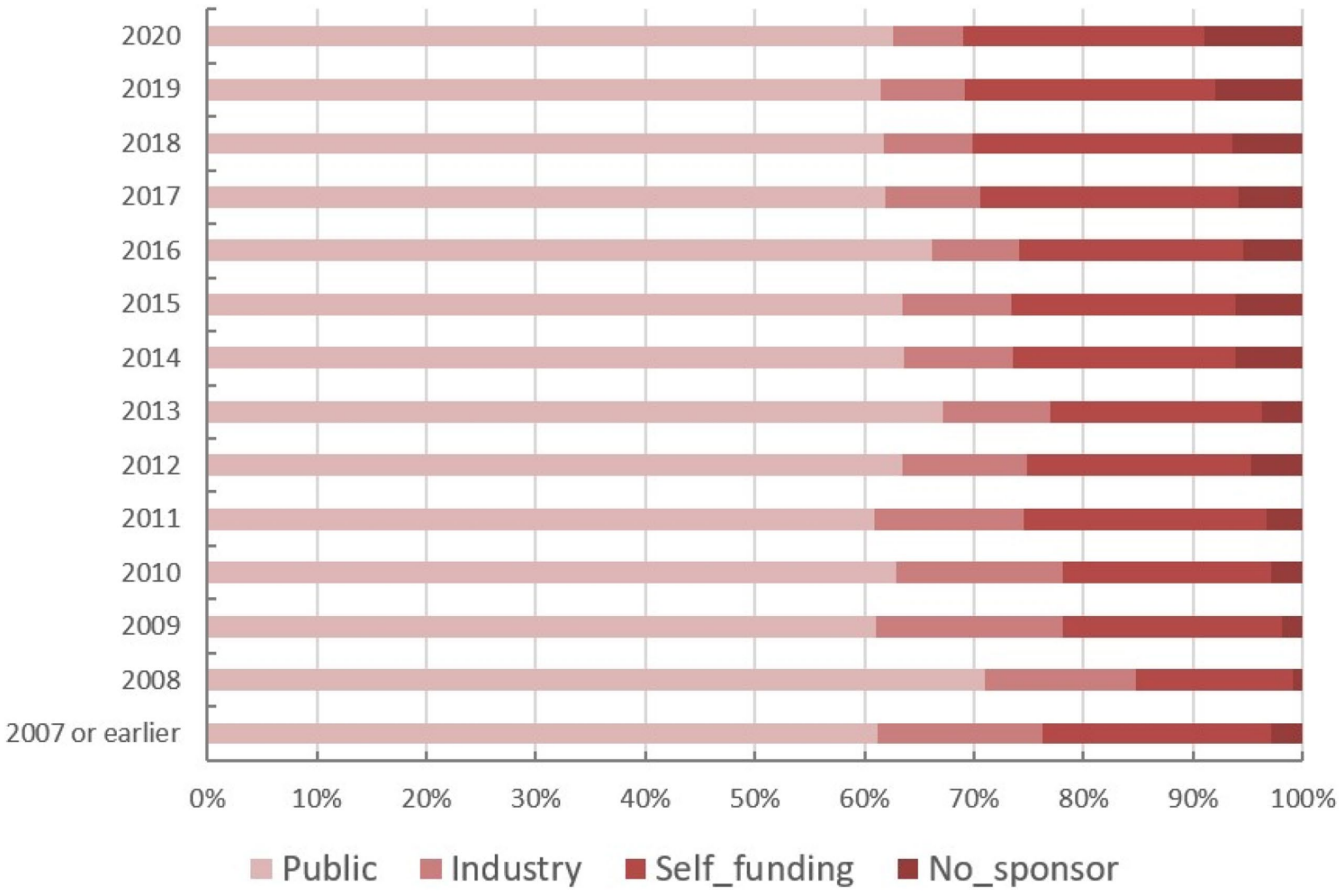
The annual change of proportion for trials sponsored by each funding type from 2007 to 2020.

The funding source of registered trials was also analyzed. Trials supported by public funds, like the Natural Science Foundation of China (NSFC), non-profit organizations, the National Special Plan for the Development of Science and Technology, and others, composed the largest part (19,828 trials, 62%) (Figure 9), followed by self-funded (6,935 trials, 22%), and industry-funded trials (2,691 trials, 8%). The overall proportion for each type of funding was depicted in Figure 9. The annual change of proportion for trials sponsored by each funding type from 2007 to 2020 was also depicted, public funding supported more than half of the clinical trials each year (Figure 10).

Continuous growth in trial numbers within each group of funding was observed over time with a significant upward trend from 2017 to 2018. However, the trend was relatively flat before 2013. The annual variation trend of trials with various types of funding for the top 5 disease types was shown in Figures 8A–E. Although the number of trials on ChiCTR increased significantly over the years, there was no obvious change was observed in the percentage of trials funded by each type of source from 2007 to 2020 (Figure 8F).

## Discussion

Clinical trial registration is an important indicator of advanced health research activities and a source of evidence on how to improve the current status of the healthcare system (10). Nearly tens of thousands of participators participated in clinical trials in various fields every year worldwide, and it is a moral obligation and should be a legal requirement to complete the clinical trial registration process before the initiation of clinical trial (11). The time and geographic distribution could be used to reveal the level of activity in clinical research over a period of time in a particular region. Disclosing the funding sources of clinical trials is essential for assessing potential biases, identifying conflicts of interest, promoting transparency, and enabling informed decision-making regarding the reliability and applicability of trial results (12). The collected data in this study represents the distribution of health science-based factors that impact the health outcomes and indicate the area that may require further attention. Our data also indicates the financial efforts tilted toward specific diseases/pathologies. The trial registry provides a platform for monitoring the major clinical activities and allows for an estimation of the benchmarking performance for different geographic regions in China. This study is the largest analysis of all clinical trial registrations in China in a 13-year duration. It delivers an overview of all clinical trial-related activities in China and generates insights into the state of clinical research in the country. Data collected in this study may be used in quality improvement projects to direct healthcare activities by advising the healthcare organizations.

During the last two decades, the prospective registration of clinical trials became standard practice (13, 14). According to a joint statement released in September 2004, by the International Committee of Medical Journal Editors (ICMJE), all the clinically directive trials should be registered on public trials registries such as *ClinicalTrials.gov*, *EU Clinical Trials Register* (EU CTR), and ChiCTR prior to enrolment of the first patient for trials to be considered for publication by medical journals (15). The reporting and summarizing of the trial-related information publicly is designed to promote systematic disclosure and monitoring of clinical trial information (16). Despite the important significance of clinical trials in the public health field, the standard registration procedures of clinical trials play a crucial role in the trial management and the audit of data reporting (7, 8, 17–19). ChiCTR was also certified as the primary registry of the World Health Organization International Clinical Trial Registration Platform in 2007. ChiCTR is responsible for the collection and management of all clinical trial records in China, and most of the registered trials’ information is publicly available on the ChiCTR website in Chinese and English (20). As of June 18, 2021, the ChiCTR system contained records for 46,469 trials, including 39,068 pre-registration trials and 7,398 supplementary trials.

ChiCTR is the third largest clinical trial registry after *ClinicalTrials.gov* and *EU Clinical Trials Register* (EU-CTR). The *ClinicalTrials.gov* is hosted by the National Library of Medicine in the United States, which is the largest registry globally and encompasses clinical trials from around the world. It serves as a comprehensive resource for both researchers and the general public and contains information on about 396,463 trials from the USA and 220 countries (assessed on 26 November 2021) (21, 22). While EU-CTR is specifically designed to register clinical trials conducted in the European Union (EU) member states and countries associated with the EU. Other than the clinical trials registered and/or developed in China, some associated trials launched outside of China are also registered on ChiCTR. All of the registries above can serve as important resources for researchers, sponsors, and the public. Each registry has its specific focus, coverage area, and regulatory requirements, making them valuable sources of information for different audiences based on their needs and geographical locations.

The current ChiCTR-based data analysis reflects the geographic trial distribution and proportions of targeted diseases and pathologies during the last 13 years. Annual variations in the number of trials in different areas indicate the changes in the clinical trial registrations over time. Most of the trials were associated with governmental funds. The highest number of trials was supported by public funds (19,828 trials, 62%) compared to other trial-supporting funding sources in China. Furthermore, many non-profit organizations, public hospitals, colleges, and universities gain research-related support from government financial organizations before conducting trials. Accordingly, the government-funded support of trials is potentially underestimated, although the current study did not aim to estimate the exact figures of governmental support for trials in ChiCTR. Providing information on funding sources promotes transparency and accountability in clinical research. Trials with adequate funding from reputable sources, such as government agencies or non-profit organizations are often perceived to be more reliable and trustworthy.

As an important characteristic, the trial duration is also taken into account in this study. According to previous studies, the duration of clinical trials may have a significant impact on various factors such as results reporting, accessibility, and traceability (23). The duration of a clinical trial provides crucial information for planning and allocating resources effectively and helps to assess its feasibility within a given timeframe (24). Besides, trials conducted over a longer duration often allow for a more comprehensive assessment of outcomes, long-term effects, and safety profiles. Longer follow-up periods could enhance the reliability and generalizability of the findings, especially in studies evaluating chronic conditions or rare diseases. In addition, we analyzed the research phase of several clinical trials in our study. However, we performed the distribution of the research phase to describe a global perspective of both the quantity and quality of clinical trial research in China (25). The current trial research analysis enables robust internal governance which aims to enhance the performance of health systems and provide an equal distribution of funds over all geographic areas in China. The analysis also aims to develop trial-related guidelines and enhance the overall trial compliance and public and sponsor-based accountability.

Compared to the previous assessment of ChiCTR data (26, 27), our analysis collected and used the largest sample size accumulated over the last 13 years. Several studies addressed the data available on ChiCTR, although the analysis was limited to only one type of disease. One of the recent reports evaluated the characteristics of COVID-19-targeting clinical trials registered in ChiCTR and compared the data with available data in ClinicalTrials.gov (26). Another study described the characteristics of ChiCTR data related to anticancer drug testing. The cancer-focusing study reported multi-dimensional aspects of clinical trial analysis linked to funding sources, types of tested drugs, and trial phases (27). Our analysis delivers complete distribution and trend characteristics useful for researchers, sponsors, and policymakers, and provides valuable insights into the registered clinical trials on ChiCTR, subsequently highlighting significant areas for improvement and potential opportunities for the trial registry.

Accurate and comprehensive clinical trial registration may help to ensure the maximum utilization of limited medical resources and minimize bias in trials (28, 29). The current ChiCTR webpage investigation indicated registration-related faults and low availability (or incomplete availability) of the required information. We found that 190 out of 32,017 trials did not report their study design, whereas 848 trials failed to report the correct year of the trial initiation. Altogether, comprehensive information about funding resources was not found for 472 trial records. Quality and integrity of information can directly affect the quality of trial assessment and authenticity of data auditing (30–32). An improved process of trial registration and monitoring is urgently needed. We understand that any incomplete or inaccurate data on the trial registry may compromise its utility. It was also noted that open assessment strategies should be implemented for the enforcement of public accountability of all trial-initiating organizations (33).

Our study has identified several limitations that should be considered. We found that ChiCTR clinical trial records do not contain information about all clinical trials in China. The trial-related inclusion criteria that were reported for the ClinicalTrials.gov database and WHO clinical trial registration platforms (34–36) analyses were not met for some trials in China. Furthermore, the incomplete information was found for trials conducted or registered before 2007. The records we have collected are relevant to the indicated time frame and may not reflect the full content of the ChiCTR database. Finally, our study is limited to all the clinical trials conducted in China, including Hong Kong and Macao Special Administrative Regions, and Taiwan province. Some of the trials registered on ChiCTR were conducted outside of China and, consequently, are outside of this study’s scope.

## Conclusion

In China, clinical trial support for oncological and cardiovascular diseases received the largest proportion of national public funding designated to stimulate progress in health and medical treatment research. Improved quality of clinical trial records was shown to greatly contribute to the effective monitoring and allocation of funding resources within the healthcare system. The data analysis ensured greater transparency of clinical trials in China and demonstrated a research distribution over geographic areas and disease categories. The optimized and regulated registration process resulted in a better data quality assessment, particularly for newly conducted trials. The growing number of trials for all disease categories reflected the escalation of clinical research activities in China. The tendency to distribute funding resources toward exceedingly populated areas with the highest incidence of oncological and cardiovascular diseases reveals the significant objective to reduce the dominating disease burden in the urban conglomerates in China. However, standardized nomenclature should be implemented to ensure consistency. The findings of this study provide a solid foundation for the future improvement of the trial system and associated public health regulations.

## Data availability statement

The original contributions presented in the study are included in the article/supplementary material, further inquiries can be directed to the corresponding authors.

## Author contributions

RF, JL, NB, and OS: concept and design. RZhao, NB, YZ, SL, XZ, CL, SH, DZ, RZ, and BG: acquisition, analysis, and interpretation of data. RF, JL, NB, OS, PM, BG, and VNN: drafting of the manuscript. JL, NB, PM, and RF: critical revision of the manuscript for important intellectual content. DZ: statistical analysis. RF: supervision. All authors contributed to the article and approved the submitted version.

## Abbreviations

ChiCTR: Chinese Clinical Trial Registry
yoy: Year-on-year
CFDA: China Food and Drug Administration
CCVD: Cardio/−cerebrovascular diseases
EU-CTR: EU Clinical Trials Register

## References

[1] Denneny C Bourne S Kolstoe SE. Registration audit of clinical trials given a favourable opinion by UK research ethics committees. BMJ Open. (2019) 9:e026840. doi: 10.1136/bmjopen-2018-026840 PMC6398673 30796130

[2] Lockshin M Katz P Yelin E. Clinical trial registration and publication of randomized controlled trials. JAMA. (2010) 303:517–8. doi: 10.1001/jama.2010.95 20145227

[3] Schwartz LM Woloshin S Zheng E Tse T Zarin DA. ClinicalTrials.gov and drugs@FDA: a comparison of results reporting for new drug approval trials. Ann Intern Med. (2016) 165:421–30. doi: 10.7326/m15-2658 27294570 PMC5028264

[4] Magee LA Menzies J. The ClinicalTrials.gov results database. N Engl J Med. (2011) 364:2169. doi: 10.1056/NEJMc1103910 21631346

[5] Goldacre B DeVito NJ Heneghan C Irving F Bacon S Fleminger J. Compliance with requirement to report results on the EU clinical trials register: cohort study and web resource. BMJ. (2018) 362:k3218. doi: 10.1136/bmj.k3218, PMID: 30209058 PMC6134801

[6] Taixiang, BZ WU Youping LI Hongcai SHANG. Promoting standardization of clinical trial data management in China. Chin J Evid Based Med. (2018) 18:532–7. doi: 10.7507/1672-2531.201804096

[7] Prayle A Hurley M Smyth A. Compliance with mandatory reporting of clinical trial results on ClinicalTrials.gov: cross sectional study. BMJ. (2012) 344:d7373. doi: 10.1136/bmj.d7373 22214756

[8] Anderson ML Chiswell K Peterson ED Tasneem A Topping J Califf RM. Compliance with results reporting at ClinicalTrials.gov. N Engl J Med. (2015) 372:1031–9. doi: 10.1056/NEJMsa1409364 25760355 PMC4508873

[9] Wyatt J. Use and sources of medical knowledge. Lancet. (1991) 338:1368–73. doi: 10.1016/0140-6736(91)92245-w 1682745

[10] Trofimova AV Bluemke DA. Prospective clinical trial registration: a prerequisite for publishing your results. Radiology. (2022) 302:1–2. doi: 10.1148/radiol.2021211967 34609194

[11] Chan AW Sim I Gülmezoglu AM Unterlerchner P Karam G Pang T. Assessing clinical trial results. Science. (2006) 312:365–6. author reply: 365–6. doi: 10.1126/science.312.5772.365b 16634140

[12] Fisher CB. Public Health. Clinical trials results databases: unanswered questions. Science. (2006) 311:180–1. doi: 10.1126/science.1119685 16410509

[13] MacCallum C Skandarajah A Gibbs P Hayes I. The value of clinical colorectal cancer registries in colorectal cancer research: a systematic review. JAMA Surg. (2018) 153:841–9. doi: 10.1001/jamasurg.2018.1635 29926104

[14] Hoque DME Kumari V Hoque M Ruseckaite R Romero L Evans SM. Impact of clinical registries on quality of patient care and clinical outcomes: a systematic review. PLoS One. (2017) 12:e0183667. doi: 10.1371/journal.pone.0183667 28886607 PMC5591016

[15] DeAngelis CD Drazen JM Frizelle FA Haug C Hoey J Horton R. Clinical trial registration. JAMA. (2004) 292:1363–4. doi: 10.1001/jama.292.11.1363 15355936

[16] Zarin DA Tse T Williams RJ Rajakannan T. The status of trial registration eleven years after the ICMJE policy. N Engl J Med. (2017) 376:383. doi: 10.1056/NEJMsr1601330 28121511 PMC5813248

[17] Dickersin K Rennie D. The evolution of trial registries and their use to assess the clinical trial enterprise. JAMA. (2012) 307:1861–4. doi: 10.1001/jama.2012.4230 22550202

[18] Riveros C Dechartres A Perrodeau E Haneef R Boutron I Ravaud P. Timing and completeness of trial results posted at ClinicalTrials.gov and published in journals. PLoS Med. (2013) 10:e1001566. doi: 10.1371/journal.pmed.1001566 24311990 PMC3849189

[19] Chan A Song F Vickers A Jefferson T Dickersin K Gøtzsche PC. Increasing value and reducing waste: addressing inaccessible research. Lancet. (2014) 383:257–66. doi: 10.1016/s0140-6736(13)62296-5, PMID: 24411650 PMC4533904

[20] Introduction to the Chinese Clinical Trial Registry (ChiCTR) Center. Available at: http://www.chictr.org.cn/about.aspx

[21] Hirsch B Califf R Cheng S Tasneem A Horton J Chiswell K. Characteristics of oncology clinical trials: insights from a systematic analysis of clinicalTrials. gov. JAMA Intern Med. (2013) 173:972–9. doi: 10.1001/jamainternmed.2013.627 23699837

[22] Zarin DA Ide NC Tse T Harlan WR West JC Lindberg DA. Issues in the registration of clinical trials. JAMA. (2007) 297:2112–20. doi: 10.1001/jama.297.19.2112 17507347

[23] Kunina H Al-Mashat A Chien JY Garhyan P Kjellsson MC. Optimization of trial duration to predict long-term HbA1c change with therapy: a pharmacometrics simulation-based evaluation. CPT Pharmacometrics Syst Pharmacol. (2022) 11:1443–57. doi: 10.1002/psp4.12854 35899461 PMC9662199

[24] Thompson PA Wright DE Counsell CE Zajicek J. Statistical analysis, trial design and duration in Alzheimer's disease clinical trials: a review. Int Psychogeriatr. (2012) 24:689–97. doi: 10.1017/s1041610211001116 21910950

[25] Wong CH Siah KW Lo AW. Estimation of clinical trial success rates and related parameters. Biostatistics. (2019) 20:273–86. doi: 10.1093/biostatistics/kxx069 29394327 PMC6409418

[26] Huang J He Y Su Q Yang J. Characteristics of COVID-19 clinical trials in China based on the registration data on ChiCTR and ClinicalTrials.gov. Drug Des Devel Ther. (2020) 14:2159–64. doi: 10.2147/dddt.S254354 PMC7266821 32581514

[27] Song M Guo H Chen H Hu H. Characteristics of anticancer drug studies registered on the Chinese clinical trial registry (ChiCTR) from 2007 to 2015. J Evid Based Med. (2016) 9:59–68. doi: 10.1111/jebm.12203 27203189

[28] Dwan K Altman DG Cresswell L Blundell M Gamble CL Williamson PR. Comparison of protocols and registry entries to published reports for randomised controlled trials. Cochrane Database Syst Rev. (2011) 2011:Mr000031. doi: 10.1002/14651858.MR000031.pub2 21249714 PMC7390503

[29] Stern JM Simes RJ. Publication bias: evidence of delayed publication in a cohort study of clinical research projects. BMJ. (1997) 315:640–5. doi: 10.1136/bmj.315.7109.640 9310565 PMC2127436

[30] Wager E Elia N. Why should clinical trials be registered? Eur J Anaesthesiol. (2014) 31:397–400. doi: 10.1097/EJA.0000000000000084 24992600

[31] Duley L Gillman A Duggan M Belson S Knox J Mcdonald AM. What are the main inefficiencies in trial conduct: a survey of UKCRC registered clinical trials units in the UK. Trials. (2018) 19:15. doi: 10.1186/s13063-017-2378-5 29310685 PMC5759880

[32] Pillamarapu M Mohan A Saberwal G. An analysis of deficiencies in the data of interventional drug trials registered with clinical trials registry - India. Trials. (2019) 20:535. doi: 10.1186/s13063-019-3592-0 31455366 PMC6712861

[33] DeVito NJ Bacon S Goldacre B. Compliance with legal requirement to report clinical trial results on ClinicalTrials. Gov: a cohort study. Lancet. (2020) 395:361–9. doi: 10.1016/S0140-6736(19)33220-9 31958402

[34] Zarin D Tse T Williams R Califf R Ide N. The ClinicalTrials.gov results database--update and key issues. N Engl J Med. (2011) 364:852–60. doi: 10.1056/NEJMsa1012065 21366476 PMC3066456

[35] Zarin D Fain K Dobbins H Tse T Williams R. 10-year update on study results submitted to ClinicalTrials.gov. N Engl J Med. (2019) 381:1966–74. doi: 10.1056/NEJMsr1907644 31722160 PMC8591666

[36] Zwierzyna M Davies M Hingorani A Hunter J. Clinical trial design and dissemination: comprehensive analysis of clinicaltrials.gov and PubMed data since 2005. BMJ. (2018) 361:k2130. doi: 10.1136/bmj.k2130 29875212 PMC5989153

